# Editorial: Epidemiological considerations in COVID-19 forecasting

**DOI:** 10.3389/fepid.2022.1119559

**Published:** 2023-01-16

**Authors:** Ruy Freitas Reis, Peter Congdon

**Affiliations:** ^1^ Instituto de Ciências Exatas, Departamento de Ciência da Computação, Universidade Federal de Juiz de Fora, Juiz de Fora, Brazil; ^2^ Pós-Graduação em Modelagem Computacional, Universidade Federal de Juiz de Fora, Juiz de Fora, Brazil; ^3^ Queen Mary University of London, London, United Kingdom

**Editorial on the Research Topic** Epidemiological considerations in COVID-19 forecasting

## 1. Epidemiological considerations in COVID-19 forecasting

In its initial epidemic phase from December 2019 to March 2020, Sars-CoV-2 infected about 800 thousand people worldwide and about 50 thousand of them died. The rapid spread of COVID-19 led the World Health Organization (WHO) to declare COVID-19 a global pandemic (1). Preliminary estimates suggest total global deaths attributable to COVID-19 throughout 2020 to be at least 3 million (2). The unusual rapid spread of a new disease led the scientific community to make a great effort to understand and represent the mechanisms underlying the spread of the pandemic.

Many mathematical and computational models have been adapted to describe the epidemiological behavior of COVID-19 spread, including predicting the dynamics to assist efforts to counter rapid dissemination of the disease (3–5). Different modeling strategies to describe the pandemic include stochastic/probabilistic (3, 6–9), and chaotic (10, 11), with many models using ODEs (Ordinary Differential Equations) adapting the compartmental SIR (Susceptible, Infected, and Recovered) model (5, 12–17). Many studies of COVID dynamics have been at national level, but spatially disaggregated approaches (e.g. spatio-temporal forecasts) have been proposed, raising questions about localized diffusion between nearby populations (18, 19).

Projecting possible scenarios of the pandemic’s duration, wave fluctuations and peaks provides valuable information for health public pandemic planning (20). Scenario planning is also relevant for economic reasons since many countries that have adopted circulation restrictions to reduce the spread of the disease still suffer from economic impacts and wider social ramifications (9). Furthermore, the use of computational tools for predicting potential high-risk areas to be monitored is also an important tool for health public strategies (21). On the other hand, following progress in developing effective vaccines many researchers have attempted to describe mathematically the impact of alternative vaccination strategies on viral spread dynamics (9, 14, 22).

## 2. Survey of papers in this research topic

About the time the COVID-19 pandemic started, the Global Health Security Index (GHSI) was published. The GHSI was proposed to score countries’ preparedness for a pandemic. A few months after the start of the pandemic, researchers began to analyze the validity of the GHSI. They correlated national COVID per capita death rates with GHSI scores. Surprisingly, they showed that the better prepared a country, the higher the death rate, i.e. a result that was counter to what would have been expected. Goldschmidt et al. takes another look at the GHSI by exploring the relationship in major European Union countries plus the United Kingdom.

Managing the COVID-19 pandemic continues to be a challenge due to poor adherence to COVID-19 prevention measures worldwide. The study of Eyeberu et al. aims to identify the determinants of community adherence to pandemic prevention among adults in the Harari Regional State of Eastern Ethiopia. They discovered that about half of the study participants showed poor adherence. On the other hand, pandemic management also requires appropriate and timely measures by government and non-governmental organizations.

Before applying diagnostic tests for screening purposes it is important to understand the baseline risk in the tested population. Particularly, in the COVID-19 pandemic, the incidence rate remains to change. The study of McAloon et al. uses incidence data to estimate the prevalence of community infection at two particular points in time. Their proposed methodology has the potential as a real-time estimation to support decision-making regarding control measures needed to allow mass gatherings while the pandemic is still to some degree extant. The WHO emphasize the importance of guidance for enabling mass gatherings (23).

The study of Lohia et al. analyses the epidemiological importance of testing the Indian population for COVID-19 during the pandemic. This research work is a retrospective analysis of the testing data collected by the Indian Council of Medical Research (about 170 million tests up to December 29, 2020). This study aimed to understand the probability of a person testing negative after an initial positive test and to evaluate the varied impact and duration of the disease in people of different age groups and genders.
